# Impact of resection for ovarian metastases from colorectal cancer and clinicopathologic analysis: A multicenter retrospective study in Japan

**DOI:** 10.1002/ags3.12740

**Published:** 2023-09-14

**Authors:** Hiroyasu Kagawa, Yusuke Kinugasa, Tomohiro Yamaguchi, Masayuki Ohue, Kazushige Kawai, Junichiro Hiro, Seiichi Shinji, Hiroaki Nozawa, Yasumitsu Hirano, Koji Komori, Yasumasa Takii, Takeshi Suto, Shunsuke Tsukamoto, Yoshito Akagi, Heita Ozawa, Yuji Toiyama, Kazuhito Minami, Tomoharu Shimizu, Kay Uehara, Kazuhiro Sakamoto, Keita Mori, Kenichi Sugihara, Yoichi Ajioka

**Affiliations:** ^1^ Study Group for Ovary Metastasis from Colorectal Cancer by the Japanese Society for Cancer of the Colon and Rectum; ^2^ Division of Colon and Rectal Surgery Shizuoka Cancer Center Shizuoka Japan; ^3^ Department of Gastrointestinal Surgery Tokyo Medical and Dental University Tokyo Japan; ^4^ Department of Gastroenterological Surgery Cancer Institute Hospital of Japanese Foundation for Cancer Research Tokyo Japan; ^5^ Department of Gastroenterological Surgery Osaka International Cancer Institute Osaka Japan; ^6^ Department of Surgery Tokyo Metropolitan Cancer and Infectious Diseases Center Komagome Hospital Tokyo Japan; ^7^ Department of Surgery Fujita Health University Hospital Toyoake Japan; ^8^ Department of Gastrointestinal Hepato‐Biliary‐Pancreatic Surgery Nippon Medical School Tokyo Japan; ^9^ Department of Surgical Oncology, Faculty of Medicine The University of Tokyo Tokyo Japan; ^10^ Department of Gastroenterological Surgery Saitama Medical University International Medical Center Saitama Japan; ^11^ Department of Gastroenterological Surgery Aichi Cancer Center Hospital Nagoya Japan; ^12^ Department of Gastroenterological Surgery Niigata Cancer Center Hospital Niigata Japan; ^13^ Department of Gastroenterological Surgery Yamagata Prefectural Central Hospital Yamagata Japan; ^14^ Department of Colorectal Surgery National Cancer Center Hospital Tokyo Japan; ^15^ Department of Surgery Kurume University School of Medicine Kurume Japan; ^16^ Department of Surgery Tochigi Cancer Center Tochigi Japan; ^17^ Department of Gastrointestinal and Pediatric Surgery, Division of Reparative Medicine, Institute of Life Sciences Mie University Graduate School of Medicine Tsu Japan; ^18^ Department of Surgery Matsuyama Red Cross Hospital Matsuyama Japan; ^19^ Division of Medical Safety Section Shiga University of Medical Science Otsu Japan; ^20^ Division of Surgical Oncology, Department of Surgery Nagoya University Graduate School of Medicine Nagoya Japan; ^21^ Department of Coloproctological Surgery Juntendo University Faculty of Medicine Tokyo Japan; ^22^ Department of Biostatistics, Clinical Research Center Shizuoka Cancer Center Shizuoka Japan; ^23^ Tokyo Medical and Dental University Tokyo Japan; ^24^ Division of Molecular and Diagnostic Pathology, Graduate School of Medical and Dental Sciences Niigata University Niigata Japan

**Keywords:** colorectal cancer, ovarian metastasis, ovarian resection, peritoneal metastasis, prognostic factor

## Abstract

**Aim:**

The aim of this study was to clarify the significance of resection of ovarian metastases from colorectal cancer and to identify the clinicopathologic characteristics.

**Methods:**

In this multicenter retrospective study, we evaluated data on ovarian metastases from colorectal cancer obtained from patients at 20 centers in Japan between 2000 and 2014. We examined the impact of resection on the prognosis of patients with ovarian metastases and examined prognostic factors.

**Results:**

The study included 296 patients with ovarian metastasis. The 3‐y overall survival rate was 68.6% for solitary ovarian metastases. In all cases of this cohort, the 3‐y overall survival rates after curative resection, noncurative resection, and nonresection were 65.9%, 31.8%, and 6.1%, respectively (curative resection vs noncurative resection [*P <* 0.01] and noncurative resection vs nonresection [*P <* 0.01]). In the multivariate analysis of prognostic factors, tumor size of ovarian metastasis (*P <* 0.01), bilateral ovarian metastasis (*P =* 0.01), peritoneal metastasis (*P <* 0.01), pulmonary metastasis (*P =* 0.04), liver metastasis (*P <* 0.01), and remnant of ovarian metastasis (*P <* 0.01) were statistically significantly different.

**Conclusion:**

The prognosis after curative resection for solitary ovarian metastases was shown to be relatively favorable as Stage IV colorectal cancer. Resection of ovarian metastases, not only curative resection but also noncurative resection, confers a survival benefit. Prognostic factors were large ovarian metastases, bilateral ovarian metastases, the presence of extraovarian metastases, and remnant ovarian metastases.

## Graphical abstract

Resection of ovarian metastases from colorectal cancer, not only curative resection but also noncurative resection, confers a survival benefit. Prognostic factors were large ovarian metastases, bilateral ovarian metastases, the presence of extraovarian metastases, and remnant ovarian metastases.

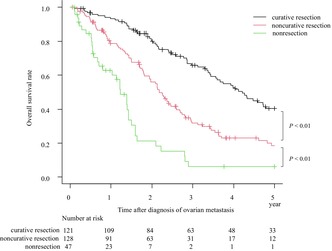

## 1 INTRODUCTION

According to Global Cancer Statistics 2020, the number of new cases of colorectal cancer (CRC) worldwide is 1.88 million, ranking third after breast cancer and lung cancer. In addition, the number of deaths from CRC is 920,000, ranking second after lung cancer.^1^ Approximately 1.1%–4.2% of women with CRC are diagnosed with ovarian metastasis,^2^, ^3^ and median survival for these women ranges from 19 to 27 mo.^4^, ^5^

In recent years, chemotherapy including molecular‐targeted therapy has evolved, improving the survival time for metastatic CRC. However, ovarian metastases are less responsive to chemotherapy than other sites, and the ovary is characterized as a “sanctuary for metastases.”^6^ Surgical resection should always be considered in cases of ovarian metastases, even in the presence of extraovarian metastases.

The pathway leading from CRC to ovarian metastasis remains unclear. Several theoretical routes that may involve direct invasion, peritoneal dissemination,^7^ hematogenous metastasis, or lymphatic metastasis.^8^, ^9^ Ovarian metastasis is currently classified as distant metastasis (M) according to both the Japanese Classification of Colorectal, Appendiceal, and Anal Carcinoma (JCCRC),^10^ and TNM classification systems, although the Japanese classification system previously classified it as peritoneal metastasis.

Previous reports have analyzed the prognosis, prognostic factor, treatment, and characteristics by small sample sizes at a single institution. However, cases with ovarian metastases are often accompanied by other distant metastases, and robust data on clinicopathologic characteristics, prognosis, and the effect of treatment are lacking. Consequently, no consensus on the management of ovarian metastases from CRC worldwide exists. The significance of surgery for ovarian metastases and treatment strategies, including prophylactic and palliative ovarian resection, are controversial.

This study was conducted as a multicenter and retrospective study to clarify the clinicopathological features, prognosis, and significance of ovarian resection for ovarian metastasis from CRC.

## 2 METHODS

### 2.1 Study design

This retrospective study evaluated data from patients at 20 institutions in Japan. The study protocol was approved by Ethics Committees of the Japanese Society for Cancer of the Colon and Rectum (JSCCR) and by the Institutional Review Boards of each participating hospital. The protocol of this study conforms to the provisions of the Declaration of Helsinki. Each patient provided informed consent for the use of their data, in accordance with the institutional regulations of the respective participating institution. The protocol of this study was registered at the University Hospital Medical Information Network Clinical Trial Registry (UMIN000029371).

### 2.2 Patients and methods

The study included patients diagnosed with ovarian metastasis from CRC between 2000 and 2014 from the 20 Japanese institutions. Primary CRC was histologically diagnosed as adenocarcinoma. The diagnosis of ovarian metastasis was based on histological findings in cases that involved ovarian resection, or imaging findings (e.g. computed tomography, positron emission tomography, and magnetic resonance imaging) in cases that did not resect ovarian metastasis. Cases were excluded if they involved oophorectomy for other diseases, the previous treatment of other pelvic malignancies, or appendix cancer.

Patient data were collected retrospectively from medical records containing the following information: age, primary tumor site, histology, TNM classification, site of distant metastasis, vascular invasion, lymphovascular invasion, preoperative serum carcinoembryonic antigen level, preoperative serum carbohydrate antigen 19‐9 level, resection of primary tumor, curability according to JCCRC,^10^ size of ovarian tumor, timing of ovarian metastasis, unilateral or bilateral ovarian metastasis, rupture of the ovarian tumor, treatment for the ovarian tumor, unilateral or bilateral oophorectomy, intention to undergo surgical treatment, postoperative chemotherapy, recurrence after resection of ovarian metastasis, and survival interval.

The primary outcome assessed was survival after diagnosis of the ovarian metastasis, which was evaluated according to curative resection, noncurative resection, or nonresection of the ovarian metastasis. The secondary outcomes assessed included prognostic factors for ovarian metastasis in CRC, the clinicopathological characteristics of ovarian metastasis from CRC, and survival of solitary ovarian metastases.

### 2.3 Definition

The overall survival (OS) period was defined as the survival period after surgery in cases with ovarian resection and the survival period from the date of imaging diagnosis in cases without ovarian resection. Synchronous metastases were defined as those diagnosed at the same time as the primary tumor, and metachronous metastases were defined as those diagnosed after the primary tumor had been resected.

The definition of curability for primary tumors is defined as follows in accordance with the JCCRC: Curability A (CurA): No distant metastasis (M0), and no residual tumor at both proximal/distal and radial margins; Curability B (CurB): Not corresponding to Curability A or C; Curability C (CurC): Macroscopic residual tumor.

In the resection of ovarian metastases, curative resection is defined as R0. Noncurative resection describes surgery for the symptomatic management of enlarged ovaries or cytoreduction, even though distant metastases at other sites may remain. Nonresection referred to ovarian metastases that had not been resected. Patients with nonresected ovaries were treated with chemotherapy, whereas those with remnant ovarian metastases were not administered any treatment. Remnant ovarian metastases, which were defined as unresected ovarian metastases, included cases in the nonresection group.

### 2.4 Statistical analysis

Continuous data are shown as median (range), and categorical data as number (%). Any differences between continuous and categorical variables were determined using the Mann–Whitney *U* test and the chi‐square test or Fisher's exact test, respectively. The OS interval was calculated based on the duration from the diagnosis of an ovarian metastasis (whether synchronous or metachronous) to the date of death due to any cause. Survival curves were estimated using the Kaplan–Meier method and compared using the log‐rank test. Variables deemed to be significant in the univariate analyses were included in a Cox proportional hazards model, and the results were expressed as hazard ratios (HRs) and 95% confidence intervals (CIs). *P* values <0.05 were significantly different. All statistical analyses were conducted using EZR (Saitama Medical Center, Jichi Medical University, Saitama, Japan), a graphical user interface for R (The R Foundation for Statistical Computing, Vienna, Austria, v. 2.13.0).

## 3 RESULTS

### 3.1 Patient characteristics

Between 2000 and 2014, a total of 20,841 female patients underwent surgery for CRC at the 20 institutions. Among these patients, 296 cases of ovarian metastasis from CRC were identified, which indicates an estimated incidence of 1.4%. A total of 116 cases of synchronous ovarian metastasis and 180 cases of metachronous ovarian metastasis exists.

The characteristics of the primary tumors of the patients who participated in this study are presented in Table 1. The median age of the study population was 55 y (range, 22–91 y). Regarding the primary site, ~91.1% of CRC patients with ovarian metastases had colon cancer or rectosigmoid cancer. The incidence of ovarian metastasis from rectal cancer was only 8.9%. Histological findings identified differentiated adenocarcinoma in 242 cases (81.8%), and undifferentiated adenocarcinoma in 36 cases (12.2%). The primary tumor was classified as T3/T4 in 269 cases (90.9%) and T1/T2 in six cases (2.0%). The lymph node metastasis from primary CRC was detected in 207 patients (69.9%). In synchronous ovarian metastases, all patients were Stage IV because of the presence of ovarian metastases. In the case of synchronous ovarian metastasis, 81 patients (69.8%) had peritoneal metastasis. Meanwhile, 34 patients (18.9%) had peritoneal metastasis in cases of metachronous ovarian metastasis. In the 73 cases of synchronous ovarian metastases (62.9%), the primary tumor was noncurative resected.

**TABLE 1:** Characteristics of primary tumor

Variable	All cases *n* = 296		Synchronous OM *n* = 116		Metachronous OM *n* = 180		*P* value
	*n*	%	*n*	%	*n*	%	
Age (years‐old)							
Median (range)	55 (22–91)		55 (24–91)		56 (22–87)		0.45
Primary tumor site							
Cecum	30	10.3	20	17.4	10	5.6	0.02
Ascending colon	46	15.8	18	15.7	28	15.8	
Transverse colon	39	13.4	10	8.7	29	16.4	
Descending colon	14	4.8	5	4.3	9	5.1	
Sigmoid colon	105	36.0	43	37.4	62	35.0	
Rectosigmoid	32	11.0	13	11.3	19	10.7	
Rectum	26	8.9	6	5.2	20	11.3	
Histology							
Differentiated type	242	81.8	100	86.2	142	78.9	<0.01
Undifferentiated type	36	12.2	15	12.9	21	11.7	
Unknown	18	6.1	1	0.9	17	9.4	
Preoperative CEA (ng/dL)							
Median (range)	20.5 (0.5–3697)		44.2 (0.5–3473)		7.4 (0.8–3697)		<0.01
Preoperative CA 19‐9 (U/mL)							
Median (range)	45 (0–18 750)		50.0 (0–18 750)		20.5 (0–7163)		<0.01
Depth of invasion							
T1	3	1.0	1	0.9	2	1.1	0.28
T2	3	1.0	0	0.0	3	1.7	
T3	77	26.0	24	20.7	53	29.4	
T4	192	64.9	82	70.7	110	61.1	
Unknown	21	7.1	9	7.8	12	6.7	
Lymph node metastasis							
N(−)	44	14.9	14	12.1	30	16.7	0.37
N(+)	207	69.9	81	69.8	126	70.0	
Unknown	45	15.2	21	18.1	24	13.3	
Cytology of ascites							
Positive	40	13.5	24	20.7	16	8.9	<0.01
Negative	93	31.4	39	33.6	54	30.0	
Unknown	163	55.1	53	45.7	110	61.1	
Stage							
I	2	0.7	0	0.0	2	1.1	<0.01
II	25	8.4	0	0.0	25	13.9	
III	82	27.7	0	0.0	82	45.6	
IV	184	62.2	116	100.0	68	37.8	
Unknown	3	1.0	0	0.0	3	1.7	
Distant metastasis site							
Ovary	116	39.2	116	100.0	0	0.0	<0.01
Peritoneum	115	38.9	81	69.8	34	18.9	<0.01
Liver	69	23.3	34	29.3	35	19.4	0.11
Lung	28	9.5	18	15.5	10	5.6	0.01
Distant lymph node	20	6.8	12	10.3	8	4.4	0.12
Other	2	0.7	1	0.9	1	0.6	1.00
Curability of primary tumor							
A	111	37.5	0	0.0	111	61.7	<0.01
B	77	26.0	43	37.1	34	18.9	
C	108	36.5	73	62.9	35	19.4	

Abbreviations: CA 19‐9, carbohydrate antigen 19‐9; CEA, carcinoembryonic antigen; OM, ovarian metastasis.

The characteristics of ovarian metastases from CRC are presented in Table 2. Bilateral metastases were found in 112 patients (37.8%) and unilateral metastases in 184 patients (62.2%). The median tumor diameter of ovarian metastases was 10 cm. The median disease‐free interval duration after primary resection of metachronous ovarian metastasis was 13.1 mo. Solitary ovarian metastasis occurred only in 22 cases (19.0%) of synchronous ovarian metastasis, while 66 cases (36.7%) of metachronous ovarian metastasis occurred (*P <* 0.01). Bilateral oophorectomy was performed in 191 patients (64.5%). Resection of the ovarian metastasis was performed in 252 patients (85.1%), with surgeries classified as curative resection in 121 cases (40.9%) and noncurative resection in 128 cases (43.2%).

**TABLE 2:** Characteristics of ovary metastasis

Variable	All cases *n* = 296		Synchronous OM *n* = 116		Metachronous OM *n* = 180		*P* value
	*n*	%	*n*	%	*n*	%	
Ovarian involvement							
Unilateral	184	62.2	64	55.2	120	66.7	0.05
Bilateral	112	37.8	52	44.8	60	33.3	
Tumor size of ovary (cm)							
Median (range)	10 (1.3–30)		10 (1.8–30)		10 (1.3–30)		0.62
Rupture of ovarian tumor							
Yes	25	8.4	12	10.3	13	7.2	0.40
No	238	80.4	94	81.0	144	80.0	
Unknown	33	11.1	10	8.6	23	12.8	
Disease‐free interval (mo)							
Median (range)					13.1 (2.6–91.9)		
Solitary ovarian metastasis							
Yes	88	29.7	22	19.0	66	36.7	<0.01
No	208	70.3	94	81.0	114	63.3	
Other distant metastasis with OM							
Peritoneum	146	49.3	81	69.8	65	36.1	<0.01
Liver	66	22.3	34	29.3	32	17.8	0.02
Lung	45	15.2	18	15.5	27	15.0	1.00
Distant lymph node	31	10.5	12	10.3	19	10.6	1.00
Other	9	3.0	1	0.9	8	4.4	0.09
Numbers of distant metastasis site							
1	88	29.7	22	19.0	66	36.7	<0.01
2	140	47.3	58	50.0	82	45.6	
3	50	16.9	23	19.8	27	15.0	
4	15	5.1	10	8.6	5	2.8	
5	3	1.0	3	2.6	0	0.0	
Treatment of ovarian metastasis							
Surgery	252	85.1	103	88.8	149	82.8	0.10
Chemotherapy	38	12.8	9	7.8	29	16.1	
Nontreatment	9	3.0	4	3.4	5	2.8	
Surgical procedure							
Unilateral oophorectomy	58	19.6	31	26.7	27	15.0	0.04
Bilateral oophorectomy	191	64.5	72	62.1	119	66.1	
Curability of surgical treatment							
Curative resection	121	40.9	40	34.5	81	45.0	<0.01
Noncurative resection	128	43.2	63	54.3	65	36.1	
Nonresection	47	15.9	13	11.2	34	18.9	
Postoperative adjuvant chemotherapy after curative resection							
Yes	74	61.2	25	62.5	49	60.5	0.21
No	47	38.8	15	37.5	32	39.5	

Abbreviations: OM, ovarian metastasis.

### 3.2 Oncological outcomes

**FIGURE 1:**
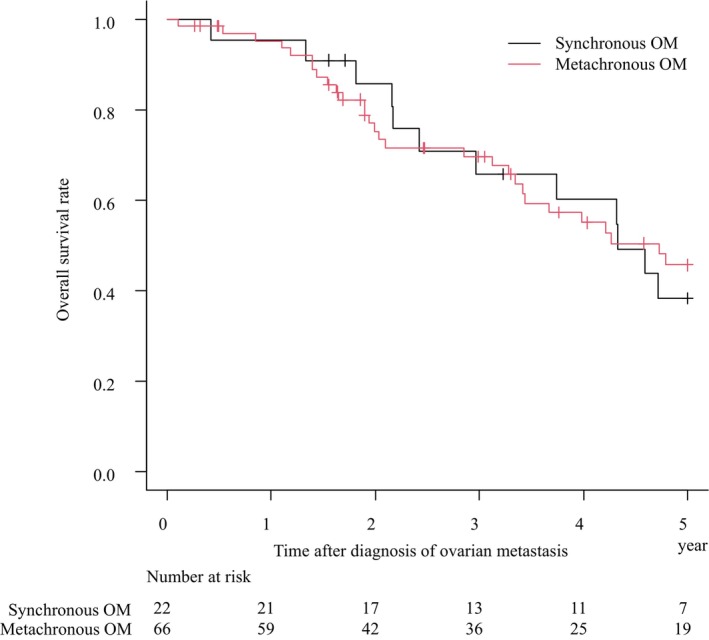
Overall survival of solitary ovarian metastasis. Overall survival curves are shown for patients with synchronous OM and metachronous OM. Black line: Synchronous OM, Red line: Metachronous OM. OM: ovary metastasis.

**FIGURE 2:**
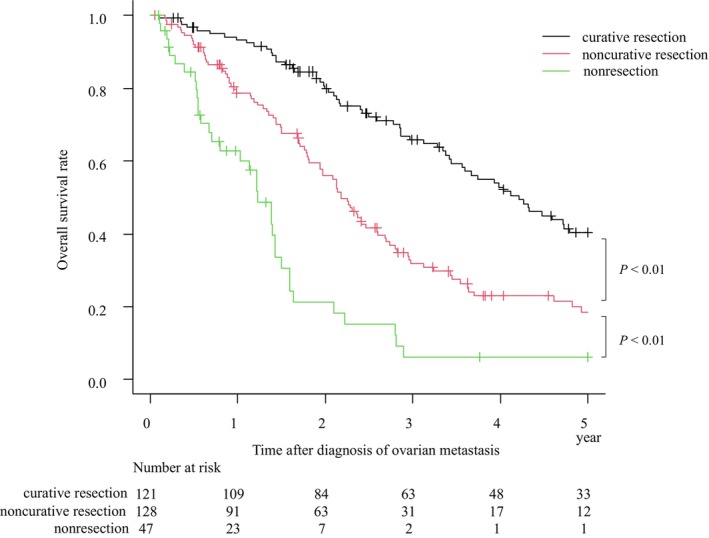
Overall survival after curative resection, noncurative resection, and nonresection. Overall survival curves are shown for patients in the curative resection group, noncurative resection group, and nonresection group. Black line: Curative resection, Red line: Noncurative resection, Green line: Nonresection. Significant differences were observed in the survival curves between the curative resection group vs noncurative resection group (*P <* 0.01, log‐rank), and in the noncurative resection group vs nonresection group (*P <* 0.01, Log rank).

The median duration of follow‐up of the study population was 36.6 mo (range: 1–201 mo). The 3‐y OS rate for solitary ovarian metastasis was 68.6% (95% CI: 57.1%–77.6%), median survival time (MST): 55.1 mo, and for synchronous and metachronous ovarian metastasis, 65.7% (95% CI: 41.0%–82.0%), MST: 52.0 mo, and 69.7% (95% CI: 56.2%–79.8%), MST: 56.7 mo, respectively (Figure 1). In all cases of this study, the 3‐y OS rates were 65.9% (95% CI: 56.0%–74.1%), MST: 50.6 mo for curative resection, 31.8% (95% CI: 23.3%–40.6%), MST: 26.2 mo for noncurative resection, and 6.1% (95% CI: 1.1%–17.6%), MST: 14.7 mo for nonresection (Figure 2). Significant differences were observed in the survival curves between the curative resection group vs noncurative resection group (*P <* 0.001), and in the noncurative resection group vs nonresection group (*P <* 0.001). The background of patients according to the curability of surgical treatment is shown in Table 3. The clinicopathological background of the curative resection group was characterized by more patients with metachronous metastasis, tumor size <10 cm, unilateral metastases, and less frequent metastases at other distant metastasis site (in comparison with the noncurative resection group). The clinicopathological background of the noncurative resection group was characterized by more patients aged <55 y, synchronous metastases, and tumor size ≥10 cm (in comparison with the non resection group). However, compared to nonresection group, no significant differences in primary tumor depth of invasion, lymph node metastasis, and frequency of distant metastases were observed.

**TABLE 3:** Patient characteristics categorized by the curability of surgical treatment

Variable	All cases *n* = 296		Curative resection (CR) *n* = 121		Noncurative resection (NCR) *n* = 128		Nonresection (NR) *n* = 47		CR vs NCR *P* value	NCR vs NR *P* value	CR vs NR *P* value
	*n*	%	*n*	%	*n*	%	*n*	%			
Age											
<55	154	52.0	68	56.2	70	54.7	16	34.0	0.06	0.02	0.01
55≤	142	48.0	53	43.8	58	45.3	31	66.0			
Timing of ovary metastasis											
Synchronous	116	39.2	40	33.1	63	49.2	13	27.7	0.01	0.01	0.50
Metachronous	180	60.8	81	66.9	65	50.8	34	72.3			
Location											
Right	115	38.9	41	33.9	55	43.0	19	40.4	0.14	0.76	0.42
Left	181	61.1	80	66.1	73	57.0	28	59.6			
Histology											
Differentiated type	242	81.8	102	84.3	107	83.6	33	70.2	0.46	0.10	0.10
Undifferentiated type	36	12.2	11	9.1	16	12.5	9	19.1			
Unknown	18	6.1	8	6.6	5	3.9	5	10.6			
Depth of invasion											
T1–T3	102	34.5	42	34.7	40	31.3	20	42.6	0.50	0.16	0.38
T4	192	64.9	77	63.6	88	68.8	27	57.4			
Lymph node metastasis											
N(−)	44	14.9	24	19.8	15	11.7	5	10.6	0.12	0.52	0.84
N(+)	204	68.9	88	72.7	95	74.2	21	44.7			
Distant metastasis site											
Peritoneum	146	49.3	29	24.0	86	67.2	31	66.0	<0.01	0.88	<0.01
Liver	66	22.3	6	5.0	43	33.6	17	36.2	<0.01	0.75	<0.01
Lung	45	15.2	1	0.8	29	22.7	15	31.9	<0.01	0.21	<0.01
Distant lymph node	31	10.5	1	0.8	20	15.6	10	21.3	<0.01	0.38	<0.01
Other	9	3.0	2	1.7	5	3.9	2	4.3	0.28	0.91	0.32
Numbers of distant metastasis site											
≤2	228	77.0	119	98.3	84	65.6	25	53.2	<0.01	0.13	<0.01
3≤	68	23.0	2	1.7	44	34.4	22	46.8			
Tumor size of ovary (cm)											
<10	140	47.3	63	52.1	48	37.5	29	61.7	0.03	0.01	0.37
10≤	130	43.9	48	39.7	66	51.6	16	34.0			
Ovarian involvement											
Unilateral	184	62.2	88	72.7	68	53.1	28	59.6	<0.01	0.45	0.10
Bilateral	112	37.8	33	27.3	60	46.9	19	40.4			
Rupture of ovarian tumor											
Yes	25	8.4	12	9.9	12	9.4	1	2.1	0.91	0.86	0.23
No	238	80.4	101	83.5	109	85.2	28	59.6			
Unknown	33	11.1	8	6.6	7	5.5	18	38.3			
Period											
2000–2007	99	33.4	46	38.0	39	30.5	14	29.8	0.20	0.93	0.31
2008–2014	197	66.6	75	62.0	89	69.5	33	70.2			

Table 4 shows the recurrence sites in cases of curative resection (*n* = 121) of the metastases is commonly found in the peritoneum (47 cases, 38.8%), followed by the liver (25 cases, 20.6%) and the lung (24 cases, 19.8%). Among the 28 patients who underwent unilateral oophorectomy, recurrence occurred in the contralateral ovary in five cases (17.9%).

**TABLE 4:** Recurrence site after curative resection for ovarian metastasis

Recurrence site	No. of patients	
	(*n* = 121)	
Peritoneum	47	38.8%
Liver	25	20.6%
Lung	24	19.8%
Extraregional lymph node	11	9.1%
Ovary	5^a^	4.1%
Others	14	11.6%

^a^ 17.9% in 28 patients who underwent unilateral oophorectomy.

### 3.3 Prognostic factors

The results of the univariate and multivariate analyses are presented in Table 5. Significant prognostic factors identified in the univariate analyses included lymph node metastasis (*P =* 0.01), carbohydrate antigen 19‐9 (CA 19‐9) concentration (*P <* 0.01), tumor size of ovarian metastasis (*P =* 0.02), bilateral ovarian metastasis (*P <* 0.01), peritoneal metastasis (*P <* 0.01), pulmonary metastasis (*P <* 0.01), liver metastasis (*P <* 0.01), distant lymph node metastasis (*P <* 0.01), and remnant of ovarian metastasis (*P <* 0.01). The multivariate analysis confirmed that shorter survival was independently predicted by tumor size of ovarian metastasis (HR: 1.800; 95% CI: 1.231–2.632; *P <* 0.01), bilateral ovarian metastasis (HR: 1.657, 95% CI: 1.105–2.484; *P =* 0.01), peritoneal metastasis (HR: 1.681, 95% CI: 1.141–2.476; *P <* 0.01), pulmonary metastasis (HR: 1.826, 95% CI: 1.013–3.293; *P =* 0.04), liver metastasis (HR: 2.062, 95% CI: 1.323–3.214; *P <* 0.01), and remnant of ovarian metastasis (HR: 5.239, 95% CI: 2.746–9.994; *P <* 0.01).

**TABLE 5:** Prognostic factors in patients with ovarian metastasis

	Univariate analysis		Multivariate analysis	
	HR (95% CI)	*P*	HR (95% CI)	*P*
Age ≥ 55 (vs <54)	0.964 (0.728–1.278)	0.80		
Tumor location Left (vs Right)	0.906 (0.679–1.207)	0.50		
T stage T4 (vs T1–T3)	1.311 (0.957–2.195)	0.08	1.475 (0.949–2.292)	0.08
LN metastasis N+ (vs N−)	1.717 (1.117–2.639)	0.01	1.481 (0.851–2.577)	0.16
Cytology CY1 (vs CY0)	0.921 (0.577–1.468)	0.72		
CEA ≥ 5 (vs <5)	1.428 (0.996–2.041)	0.05	0.938 (0.603–1.462)	0.78
CA 19‐9 ≥ 40 (vs <40)	1.831 (1.333–2.513)	<0.01	1.487 (0.983–2.250)	0.06
OM synchronous (vs metachronous)	0.977 (0.734–1.300)	0.87		
OM rapture + (vs −)	0.990 (0.607–1.616)	0.97		
OM size ≥10 cm (vs <10 cm)	1.418 (1.054–1.907)	0.02	1.800 (1.231–2.632)	<0.01
OM bilateral (vs unilateral)	1.509 (1.132–2.011)	<0.01	1.657 (1.105–2.484)	0.01
Peritoneal metastasis + (vs −)	1.620 (1.221–2.149)		1.681 (1.141–2.476)	<0.01
Pulmonary metastasis + (vs −)	2.411 (1.669–3.484)	<0.01	1.826 (1.013–3.293)	0.04
Liver metastasis + (vs −)	2.247 (1.628–3.100)	<0.01	2.062 (1.323–3.214)	<0.01
Distant lymph node metastasis + (vs −)	2.024 (1.303–3.144)	<0.01	1.654 (0.833–3.281)	0.15
Remnant of ovarian metastasis yes (vs no)	2.382 (1.765–3.216)	<0.01	5.239 (2.746–9.994)	<0.01

Abbreviations: CA 19‐9, carbohydrate antigen 19‐9; CEA, carcinoembryonic antigen; CI, confidence interval; LN, lymph node; OM, ovarian metastasis; OR, odds ratio.

## 4 DISCUSSION

In this study, solitary ovarian metastases have a relatively good prognosis as Stage IV, and resection of ovarian metastases can prolong the prognosis, prognostic factors, and characteristics of ovarian metastases. This retrospective study examined ovarian metastases from CRC, which is the largest number of cases in previous reports, excluding the national registry study.^3^ Ovarian metastasis from CRC, which occurs relatively rarely, is often a complex condition with variable timing of development and other distant metastases; hence, investigating many cases is necessary.

In this study, solitary ovarian metastasis, whether synchronous or metachronous ovarian metastasis, is expected to have a prolonged prognosis. According to the results of this cohort, the 3‐y OS of solitary ovarian metastasis was 68.6%, which is a relatively promising prognosis among Stage IV CRC patients. Previous studies examining prognostic factors have reported extraovarian metastasis as an independent prognostic factor.^11^, ^12^, ^13^, ^14^ Because most ovarian metastases are confounded by extraovarian metastases, including peritoneal metastases, it may be the cause for the recognized poor prognosis.

Resection of ovarian metastases was found to be a significant prognostic factor, which was previously reported.^9^, ^12^, ^13^, ^14^ In this study, the noncurative group had a better prognosis than those in the nonresection group. Notably, patient backgrounds of the two groups revealed no significant difference in the frequency of other distant metastases, which is a prognostic factor. The nonresection group included more patients with older age, metachronous ovarian metastases, and ovarian metastases <10 cm in size. Bias in patient background may possibly be involved in whether the patient is in a condition to undergo oophorectomy. Verifying whether palliative surgery improves prognosis over chemotherapy is difficult. While ovarian metastasis is considered a sanctuary for metastasis, oophorectomy for ovaries resistant during chemotherapy was reported to allow continuation of chemotherapy and prolong the prognosis.^15^, ^16^ In this study cohort, subgroup analysis of prognosis for the 2000–2007 and 2008–2014 periods revealed no change in the prognosis of patients with ovarian metastasis (Figure S1). Despite improvements in systemic chemotherapy, no improvement in prognosis was observed, suggesting that ovarian metastasis is a sanctuary for metastasis. Palliative resection should be considered for ovarian metastases during chemotherapy for unresectable metastatic CRC.

The decision to perform bilateral oophorectomy in cases of unilateral ovarian metastases is an important question for the surgeon. In this study, 17.9% of patients who underwent unilateral oophorectomy had recurrence in the other ovary. In contrast, Zhou et al. reported recurrence in the contralateral ovary in 44% of cases in which unilateral oophorectomy was performed.^14^ Mori et al. reported that 56% of patients with preoperative diagnosis of unilateral ovarian metastasis had bilateral ovarian metastasis.^17^ The bilateral ovarian resection in these situations may be beneficial, considering that ovarian metastases are less sensitive to chemotherapy and may be affected by potential ovarian metastases. The American Society of Colon and Rectal Surgeons Clinical Practice Guidelines state that bilateral oophorectomy should typically be performed even if one ovary appears grossly normal for patients with suspected or known metastatic disease involving an ovary.^18^

In the TNM classification, ovarian metastasis is defined as a distant metastasis; however, ovarian metastasis was previously recognized as a phenotype of peritoneal dissemination in Japan and was classified as peritoneal metastasis in the past JCCRC. The metastatic pathways that lead to ovarian tumors remain unclear. Fujiwara et al. suggested that hematologic or lymphatic spread of malignant cells to the ovaries was the most likely pathway, based on the pathological examination of metastatic ovarian tumors.^9^ A review of the Swedish Cancer Registry stated that ovarian metastases are more frequently associated with peritoneal metastases, logically, as the peritoneum and ovaries are often considered a continuum, and ovarian metastases are rarely seen in rectal cancers due to anatomic factors such as extraperitoneal structures.^7^

In this study the discovered characteristics of ovarian metastasis were suggestive of an association with peritoneal metastasis. Approximately 50% of patients had synchronous ovarian and peritoneal metastases, and 30% of patients had positive cytology results for ascitic fluid. The primary sites of CRC causing ovarian metastasis in this study were colon and rectosigmoid cancer located in the abdominal cavity, which accounted for 90.9%. The frequency of ovarian metastasis from rectal cancer existing extraperitoneal was low. In addition, peritoneal recurrence was observed in 38.8% of the patients who underwent curative resection. Sato et al. performed a retrospective analysis of 172 patients with R0 resection of CRC with peritoneal metastasis and reported that 31.4% of these patients had peritoneal recurrence.^19^ Thus, the frequency of peritoneal metastatic recurrence after the resection of ovarian metastases is comparable to that after the resection of peritoneal metastases. Therefore, from a clinicopathological perspective, we suggest that CRC ovarian metastasis appears to be strongly associated with peritoneal metastasis.

If ovarian metastasis were to be classified as peritoneal metastasis, the following treatment outcomes of peritoneal metastasis would be compared and verified. The JCCRC defines peritoneal metastasis as follows: P0, no peritoneal metastasis; P1, metastasis localized to the adjacent peritoneum; P2, limited metastasis to the distant peritoneum; and P3, diffuse metastasis to the distant peritoneum. According to the system, higher numbers are associated with poorer prognoses, as indicated by the Study Group for Peritoneal Metastasis from Colorectal Cancer from the JSCCR. Peritoneal metastasis after R0 surgery has been associated with a 3‐y OS rate of 42.0% and a 5‐y OS rate of 36.2%, with 3‐y OS rates of 45.7% for P1 disease, 39.5% for P2 disease, and 25.0% for P3 disease.^19^ The 5‐y OS rates have been reported as 32.4% for R0 resection and 4.7% for R1/R2 resection.^20^ In the present study, curative resection of ovarian metastasis was associated with a 3‐y OS rate of 65.9%, which was better than the corresponding rate determined for patients with P1 disease after R0 resection. Therefore, assuming that ovarian metastasis is classified as peritoneal metastasis, we consider ovarian metastases to correspond to P1 disease based on oncologic outcome.

The results of our study are limited by a multicenter retrospective study. First, differences in treatment strategies for ovarian metastases between each institution occur. Missing values exist because data were collected from medical records retrospectively. However, this study is significant because it analyzed the largest number of cases of rare ovarian metastases. Second, this study did not examine chemotherapy in detail. The cohort included resectable and unresectable cases, synchronous ovarian metastases, and metachronous ovarian metastases, resulting in a wide variety of perioperative chemotherapies that were difficult to validate. The study population should be limited when investigating chemotherapy. Third, in this study we were unable to compare resection and nonresection groups for ovarian metastases with matched patient backgrounds. The indication for resection depends on the clinicopathologic background of the patient as well as the condition of individual patients, thereby highlighting the existence of selection bias. It is difficult to design a comparison study between resection and nonresection for ovarian metastases with low response rates to chemotherapy.

In conclusion, we investigated the oncologic outcome and clinicopathologic characteristics of ovarian metastases from a larger number of CRC cases in this multicenter retrospective study. The results showed that the prognosis of solitary ovarian metastases is relatively favorable in Stage IV CRC. Furthermore, resection of ovarian metastases in both curative and noncurative is beneficial for survival. Ovarian metastasis from CRC was suggested to be associated with peritoneal metastasis.

## AUTHOR CONTRIBUTIONS

All authors substantially contributed to the conception and design and acquisition of the study. HK, KM, and YK performed statistical analysis and data interpretation. HK and YK drafted the article, and all authors critically revised it for important intellectual content. All authors gave final approval for this version to be published.

## FUNDING INFORMATION

This work was supported by the Japanese Society for Cancer of Colon and Rectum.

## CONFLICT OF INTEREST STATEMENT

The authors declare no conflicts of interest for this article. Kay Uehara MD, PhD is an editorial member of the *Annals of Gastroenterological Surgery*.

## ETHICS STATEMENT

Approval of the research protocol: The study protocol was approved by the Institutional Review Boards of all participating hospitals.

Informed Consent: N/A.

Registry and the Registration No. of the study/trial: The clinical trial registration number is UMIN000029371.

Animal Studies: N/A.

## Supporting information


Figure S1.
