# Supplementary figures and images for: Impact of resection for ovarian metastases from colorectal cancer and clinicopathologic analysis: A multicenter retrospective study in Japan
Source: Ann Gastroenterol Surg. 2023 Sep 14;8(2):273–83. doi: 10.1002/ags3.12740 (PMC10914693; doi:10.1002/ags3.12740)

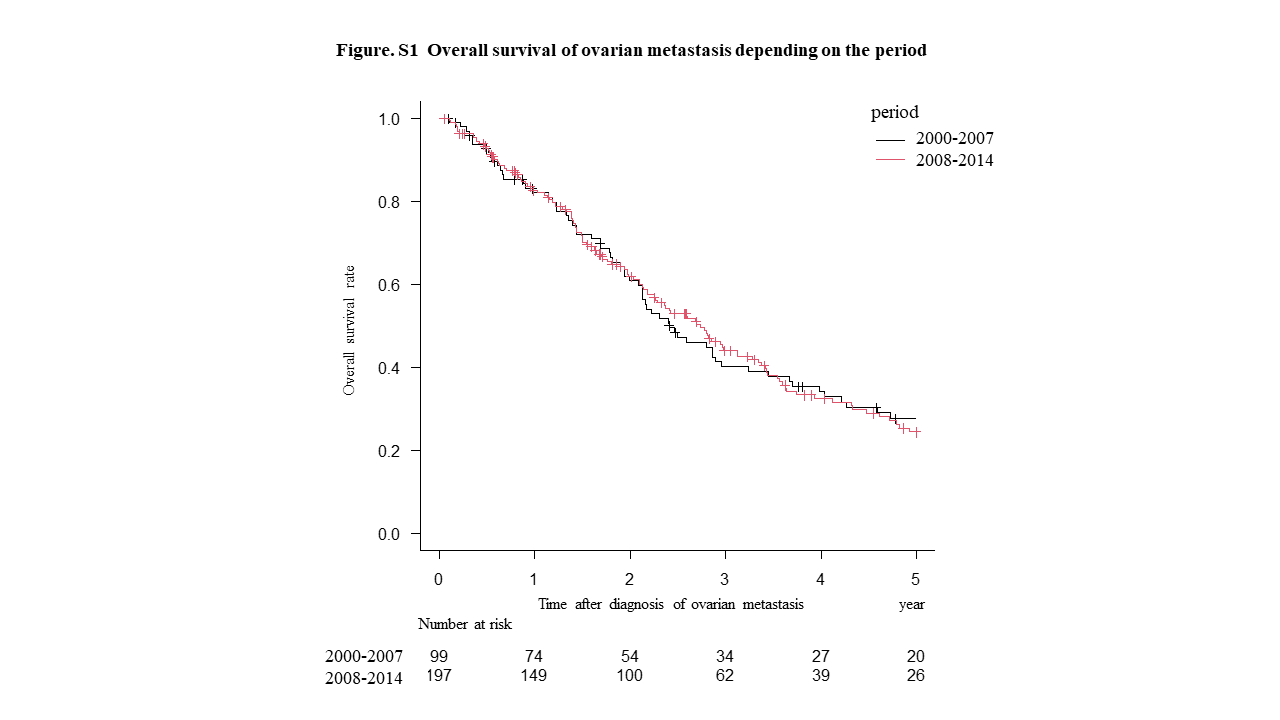

Supplement: Supplementary file 1 — Figure S1. [file AGS3-8-273-s001.tif]
